# Regulating soil bacterial diversity, community structure and enzyme activity using residues from golden apple snails

**DOI:** 10.1038/s41598-020-73184-z

**Published:** 2020-10-01

**Authors:** Jiaxin Wang, Xuening Lu, Jiaen Zhang, Guangchang Wei, Yue Xiong

**Affiliations:** 1 Department of Ecology, College of Natural Resources and Environment, South China Agricultural University, 483 Wushan Road, Tianhe District, Guangzhou, 510642 People’s Republic of China; 2 Guangdong Provincial Key Laboratory of Eco-Circular Agriculture, Guangzhou, 510642 People’s Republic of China; 3 Guangdong Engineering Research Center for Modern Eco-Agriculture and Circular Agriculture, Guangzhou, 510642 People’s Republic of China; 4 Guangdong Laboratory for Lingnan Modern Agriculture, Guangzhou, 510642 People’s Republic of China

**Keywords:** Agroecology, Microbial ecology

## Abstract

It has been shown that the golden apple snail (GAS, *Pomacea canaliculata*), which is a serious agricultural pest in Southeast Asia, can provide a soil amendment for the reversal of soil acidification and degradation. However, the impact of GAS residue (i.e., crushed, whole GAS) on soil bacterial diversity and community structure remains largely unknown. Here, a greenhouse pot experiment was conducted and 16S rRNA gene sequencing was used to measure bacterial abundance and community structure in soils amended with GAS residue and lime. The results suggest that adding GAS residue resulted in a significant variation in soil pH and nutrients (all *P* < 0.05), and resulted in a slightly alkaline (pH = 7.28–7.75) and nutrient-enriched soil, with amendment of 2.5–100 g kg^−1^ GAS residue. Soil nutrients (i.e., NO_3_-N and TN) and TOC contents were increased (by 132–912%), and some soil exocellular enzyme activities were enhanced (by 2–98%) in GAS residue amended soil, with amendment of 1.0–100 g kg^−1^ GAS residue. Bacterial OTU richness was 19% greater at the 2.5 g kg^−1^ GAS residue treatment than the control, while it was 40% and 53% lower at 100 g kg^−1^ of GAS residue and 50 g kg^−1^ of lime amended soils, respectively. Firmicutes (15–35%) was the most abundant phylum while Bacterioidetes (1–6%) was the lowest abundant one in GAS residue amended soils. RDA results suggest that the contents of soil nutrients (i.e., NO_3_-N and TN) and soil TOC explained much more of the variations of bacterial community than pH in GAS residue amended soil. Overuse of GAS residue would induce an anaerobic soil environment and reduce bacterial OTU richness. Soil nutrients and TOC rather than pH might be the main factors that are responsible for the changes of bacterial OTU richness and bacterial community structure in GAS residue amended soil.

## Introduction

Due to the greater inputs of agrochemicals and synthetic chemical fertilizers required by an increasing population^1–3^, the progression of intensive agriculture has resulted in severe soil erosion^4^, acidification^5^ and loss of fertility and biodiversity^6,7^. Anthropogenic nitrogen (N) inputs to terrestrial ecosystems have increased three to fivefold^8^ over the past century. High levels of N fertilization has caused serious soil acidification and degradation as well as environmental pollution. Guo et al.^5^ reported that the use of N fertilizer (such as ammoniacal nitrogen (NH_4_-N) and nitrate N (NO_3_-N)) may drive anthropogenic acidification, and that this acidification effect is at least 10–100 times greater than that induced by acid rain. This application and deposition of N are expected to continue to increase^9^. The most commonly used method to neutralize acidic soil is to apply Ca-amendment in the forms of either lime, gypsum or the combination of both^10^.

The invasive golden apple snail (GAS), *Pomacea canaliculata*, has become a serious problem for agricultural production in Southeast Asia^11^. A series of control methods have been developed^12,13^ and the most widely used method is chemical control by molluscicides^14^, which may unintentionally endanger environmental quality and human health^15^. Although physical and biological control methods such as manual collection or raising ducks in the paddy fields were also proposed^16,17^, the low effectiveness or high cost of these methods limited the possibility for their practical application. An alternative strategy would be to use it as an agricultural amendment. Studies have reported that GAS contains abundant CaCO_3_ and proteins and may be used as feed for livestock, such as pigs and ducks^18^. However, only small GAS can be eaten by ducks and pigs because the hard shells of large adult GAS make them unpalatable. Alternatively, a recent study suggests that GAS residue (i.e., crushed, whole GAS) can be used to neutralize acidic soils. Compared to lime, however, GAS residue also was shown to improve soil total organic carbon (TOC) and soil nutrient content^19^. Lime amendment may result in soil compaction, Si and P deficiency in soil and reduce soil microbial biomass and diversity^20^. What remains largely unknown is the effect that GAS residue has on soil microbial properties, especially bacterial community and diversity.

Previous studies have proposed that soil pH and N input are the main predictors of soil microbial diversity in soils^21,22^. However, the responses of soil microbes to elevated N inputs and pH can vary greatly. Numerous studies have revealed that N addition leads to significant reductions in soil microbial activity^23^, diversity^24^ and significant changes in community structure^25^ because of increases in carbon (C) sequestration and/or decreases in soil respiration rate^23^. Previous studies suggested that neutral soils support greater bacterial diversity than do acidic soils^21,26^. However, some studies have suggested that forest soils with lower pH support greater microbial diversity than agricultural soils with higher pH values^27^.

Soil microbes, such as bacteria and fungi, generate extracellular enzymes to release assimilable C, N and phosphorus (P) from organic compounds^28^. Soil extracellular enzymes not only play important roles in mineralization, nutrient recycling and the degradation of soil organic compounds, but they also been used as indicators of soil biological processes^29^. For example, β-1,4-glucosidase (BG), β-d-cellobiosidase (CB), β-1,4-*N*-acetylglucosaminidase (NAG) and acid phosphatase (ACP) were reported as the key enzymes associated with the recycling of nutrients related to C, N, and P^28,30^. Numerous environmental factors, such as water^31^, salinity^31^, pollution^32^, soil nutrients^33^, temperature^34^ and soil pH, can affect the activities of extracellular enzymes directly or indirectly.

In the previous study, the microbial biomass and community structure in GAS residue-amended soils were analyzed using phospholipid fatty acid (PLFA)^19^. Still, the characterization of soil bacterial communities remains unknown at a much finer taxonomic resolution, and this study aimed to achieve this through next-generation sequencing.

Here, a series of greenhouse experiments were conducted by amending GAS residue and lime to slightly acidic and degraded soils. It was hypothesized that GAS residue and lime—both of which increase soil pH—may differ in their regulation of microbial community structure, richness and microbial enzyme activity due to varying effects on soil nutrient availability and soil physiochemical properties. It was expected that the amendment of GAS residues would improve soil fertility and nutrients for microbes, consequently increase the relative abundance of some bacterial taxonomic groups, and regulate bacterial diversity. Additionally, elevated soil pH and soil nutrients induced by the amendment of GAS residue may be the major factors that are responsible for the changes of bacterial community structure and bacterial diversity.

## Materials and methods

### Testing materials

The GAS were hand-collected using butyronitrile gloves from the paddy fields at the Zengcheng Teaching and Research Farm (23° 14′ N, 113° 37′ E) of South China Agricultural University (SCAU), which is located in Zengcheng District, Guangzhou city, Guangdong Province, China. The GAS were washed and frozen at − 40 °C in a freezer for 24 h. Then the dead GAS were dried and ground into powder (GAS residues, which consisted of snail shell and meat), and packed in sealed bags and stored in a desiccator. A slightly acidic soil was also collected from the paddy fields (pH ranging from 6.25 to 6.53). The soil used in this study was the main soil type of this region where the GAS are heavily invasive and widespread. According to the USDA textural soil classification, the soil is characterized as sandy clay^35^. The soil predominately consisted of medium (37%) and fine (23%) sand, silt (5%) and clay (35%) and had 16.38 g kg^−1^ of TOC, 2.20 g kg^−1^ of total nitrogen (TN) and 0.38 g kg^−1^ of total phosphorus (TP)^19^. The GAS residue added to the soil consisted of 23.02% C, 0.58% N and 0.46% P^19^.

### Experimental design

The experiments were conducted in the Ecological Research Station (23° 09′ N, 113° 21′ E) at the SCAU campus in Guangzhou, China. Three treatments were implemented: (1) the control treatment with soil only (CK); (2) soil amended with GAS residue (SR); and (3) soil amended with lime (SL). The treated amounts of GAS residue and lime amendment were set according to the liming experiment reported by He et al.^36^. Each treatment had six levels of amendment: 0.5, 1.0, 2.5, 25, 50, and 100 g kg^−1^ (the amount in grams of GAS residue used per kg of dry soil). Low amount amendments were employed (i.e., 0.5, 1.0 and 2.5 g kg^−1^ dry weight) to explore the amendment complementarity effects of GAS residue on soil physicochemical and biological properties, and very high amounts were also employed (i.e., 25, 50 and 100 g kg^−1^) to investigate the enlarged effects of GAS residue amendment on those soil properties, as well as to explore the threshold value of overuse. Amendments were homogenized with aired soil (total weight 2 kg) for each treatment and carefully packaged in polyvinyl chloride (PVC) pots with a bottom diameter of 180 mm, a top diameter of 200 mm and a height of 260 mm. Each pot was placed on a plastic dish with a piece of filter paper beneath to prevent the loss of soil and GAS residue. At the beginning of incubation, about 600–800 mL of deionized water was poured into each pot. During the incubation period, about 400 mL of deionized water (pH = 7.0 ± 0.1) was sprayed into the pots each week to prevent the soil from drying. Each treatment was triplicated in this study. Soils were kept plant-free throughout the experiment. After 120 days of incubation^37^, the soil samples were collected and stored at 4 °C for experimental use.

### Soil analyses

Five-point sampling method was used to sample soil to the bottom of each pot using a stainless-steel soil auger (10 mm ø, 200 mm in length). And then soil samples were homogenized by hands wearing butyronitrile gloves, crushed with a rubber hammer and passed through a 2-mm sieve to remove rocks, roots and organic residues. It was then divided evenly into three subsamples using the coning and quartering technique. The three subsamples were treated as follows: the first subsample was stored at room temperature (about 22 °C) for about 4 h, the second subsample was stored at 4 °C, and the third subsample was stored at − 20 °C in preparation for further analysis^38^. The first subsample was aired at room temperature, ground, and analyzed for soil pH, TOC, TN, available phosphorus (AP), NH_4_-N and NO_3_-N. The second subsample was analyzed for soil gravimetric moisture and extracellular enzyme activity. The third subsample was lyophilized, ground into powder, and passed through a 0.25 mm sieve for DNA extraction.

Soil pH was measured from fresh soil slurries using a handheld multiparameter meter (1 g of soil: 2.5 ml of deionized water, SX-620, San Xin, China). Approximately 20 mg of each powdered sample was analyzed for the contents of TOC and TN using a Vario micro Cube elemental analyzer (Elementar, Germany). Concentrations of NH_4_-N were analyzed using a UV–Vis spectrophotometer at a wavelength of 420 nm^39^. Concentrations of NO_3_-N were determined using a UV–Vis spectrophotometer applying double wavelengths of 275 nm and 220 nm^39^. Concentrations of AP were analyzed using the molybdenum-antimony anti-spectrophotometric method^40^.

### Extracellular enzyme assay

Activities of soil enzymes, including β-1,4-glucosidase (BG), acid phosphatase (ACP), β-1,4-*N*-acetylglucosaminidase (NAG) and β-d-cellobiosidase (cellulose degradation; CB), were measured using fluorometry as described by Au-Bell et al.^41^. Incubation was conducted in a constant temperature incubator (RXZ, Dongqi, Ningbo, China) at 37 °C for 3 h. After the incubation, each sample was centrifuged (Eppendorf, USA) at 2900 rpm for 3 min, and the supernatant was pipetted and injected into a black flat-bottomed 96-well microplate for fluorescence determination in a microplate reader (SYNERGY H1, BioTek, USA) at an excitation wavelength of 365 nm and an emission wavelength of 450 nm.

### Bacterial community and diversity analysis

Soil DNA using the E.Z.N.A.® soil DNA Kit (Omega Bio-tek, Norcross, GA, USA.) according to the manufacturer’s instructions. Extracted DNA was quantified (Nanodrop 2000, Thermo Scientific, UAS), and stored − 20 °C. The universal primer sets of 338F (5′-ACTCCTACGGGAGGCAGCAG-3′) and 806R (5′-GGACTACHVGGGTWTCTAAT-3′) were used for polymerase chain reaction (PCR), which was carried out using the thermocyclingr PCR system (GeneAmp 9700, ABI, USA). PCR reactions were performed to amplify 1 μL of template DNA in a 20 μL reaction system containing 4 μL of 5 × FastPfu Buffer, 2 μL of 2.5 mM dNTPs and 0.8 μL of each primer (5 μM), 0.4 μL of FastPfu polymerase and 10 ng of template DNA. Amplification procedure was as follows: 95 °C for 3 min; 27 cyclings of 95 °C for 30 s, 30 s for annealing at 55 °C, 45 s for elongation at 72 °C and extension at 72 °C for 10 min. Each reaction was performed in triplicate, and subsequently pooled. PCR products were detected by electrophoresis in a 2% agarose gel and purified using the AxyPrepDNA Gel Extraction Kit (Axygen Biosciences, Union City, CA, U.S.) according to the manufacturer’s instructions. QuantiFluor TM-ST (Promega, USA) was used to quantify purified PCR products according to the manufacturer’s protocol. According to the standard protocol set forth by Majorbio Bio-Pharm Technology Co. Ltd. (Shanghai, China), purified amplicons were assembled in equimolar and paired-end sequences (2 × 300) on an Illumina MiSeq platform (Illumina, San Diego, USA).

Sequences were processed using QIIME (version 1.9.1) with the following criteria: (1) 250-bp reads were truncated at any site receiving an average quality score < 20 over a 10 bp sliding window, and any truncated reads shorter than 50 bp were discarded; (2) exact barcode matching, 2 nucleotide mismatch in primer matching, reads containing ambiguous characters were removed. (3) only sequences that overlap more than 10 bp were assembled according to their overlap sequence. Any reads that could not be assembled were discarded. Operational taxonomic units (OTUs) with 97% similarity using UPARSE (version 7.1 https://drive5.com/uparse/), and chimeric sequences were categorized and eliminated using UCHIME^42,43^. Each 16S rRNA gene sequence was classified using the RDP Classifier algorithm (https://rdp.cme.msu.edu/) and compared to the SILVA ribosomal RNA gene database (version 132) using a confidence threshold of 70%^44^. Prokaryotic OTUs assigned to chloroplasts and mitochondria were removed, and the resulting sequences were used for final analysis^45^. Sequence data was deposited at the NCBI SRA archive (accession number PRJNA609530).

### Statistical analyses

It should be pointed out that the data from the treatment of 100 g kg^−1^ of lime amendment was not used in analysis because the soils were overly alkalized and compacted. To evaluate the significance of GAS residue and lime amendment effects on soil physicochemical properties, Analysis of variance (ANOVA) followed by Duncan multiple comparison tests was performed using SPSS25.0 software (IBM Corp., New York, USA). When assumptions of normality and homogeneity of variance were not met, data were rank-transformed using the Rankit method to obtain normal scores for data analysis^19^. Regression analysis was performed to examine the relationship between environmental variables and bacterial OTU richness. Our assessment generated 1,889,237 high-quality 16S rRNA gene sequences from 36 samples with an average sequencing depth of 52,478 reads per sample. The sequences were clustered into 2687 OTUs and assigned to 30 phyla and 462 genera of bacteria. Each sample was subsampled to an equal sequencing depth (23,520 reads per sample, Figure S1). The variations of microbial communities were tested using ANalysis Of SIMilarity (ANOSIM) with 9999 permutations. Non-metric multidimensional scaling (NMDS) ordination was conducted to reveal the treatment effects on bacterial community structure. The Sobs index was used to assess soil bacterial OTU richness^46^. Spearman’s correlations were used to identify significant relationships between soil physical and chemical properties and the most abundant phyla and genera. Redundancy analysis (RDA) using bacterial phyla and environmental factors was performed to explore the main factors determining the bacterial community structure. Co-linearity between environmental variables was checked using variance inflation factors (VIF), and the environmental variables with VIF > 10 were removed prior to the RDA analysis.

## Results

### Variations of soil properties

**Figure 1:**
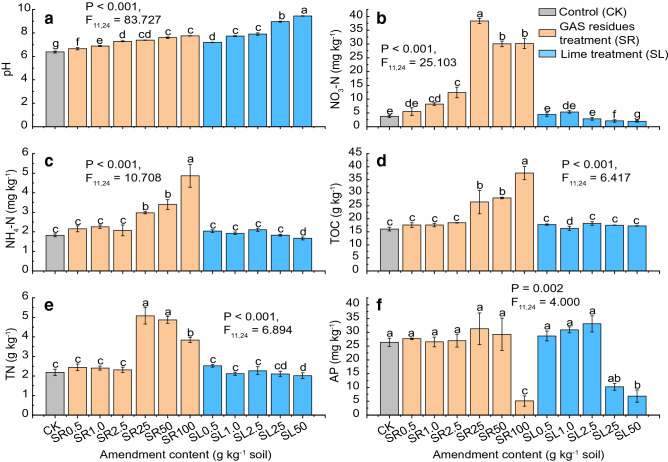
Variation in soil indicators induced by the GAS residue (SR) and lime (SL) amendments in a slightly acidic soil after incubation for 120 days. Variation in soil pH (**a**), *NO*_*3*_*-N* nitrate nitrogen, (**b**), *NH*_*4*_*-N* ammoniacal nitrogen, (**c**), *TOC* total organic carbon, (**d**), *TN* Total Nitrogen, (**e**) and *AP* Available Phosphorus, (**f**) (n = 3). Error bars represent standard errors, and the different lowercase letters indicate significance between treatments according to Duncan’s multiple range test.

Amendment of GAS residue and lime significantly increased soil pH compared with the control (*P* < 0.01, Fig. 1a). The addition of 1.0–2.5 g kg^−1^ GAS residue increased soil pH to neutral (around 7.0), and additional amendment further increased pH (up to 7.75). Even the smallest level of lime amendment (0.5 g kg^−1^) altered soil pH significantly (*P* < 0.01), from slightly acidic (6.32) to slightly alkaline (pH > 7.8) (Fig. 1a). The addition of GAS residue also increased the contents of soil carbon and soil nutrients (except for AP). Significant changes were observed at high amendment amounts (i.e*.*, 25–100 mg kg^−1^). Specifically, the contents of TOC, TN, NO_3_-N and NH_4_-N all significantly elevated with 25–100 mg kg^−1^ GAS residue amendment compared with the control (*P* < 0.05, Fig. 1b–e). The TOC and NH_4_-N progressively increased as more GAS residue was added, specifically, the levels rose by 134% (TOC) and 168% (NH_4_-N) from the control to the amendment of 100 g kg^−1^ GAS residue (all *P* < 0.01, Table S1 and Fig. 1). The contents of NO_3_-N and TN significantly increased by 912% and 132% at 25 g kg^−1^ GAS residue amendment compared with the control, but then they significantly decreased by 27% and 32% at 100 mg kg^−1^ GAS residue amendment compared with that of 25 g kg^−1^ GAS residue amendment (Fig. 1b,e). A significant decrease in AP was observed in GAS residue amendment-treated soil at the amendment amount of 100 g kg^−1^. Specifically, AP content decreased by 80% compared with the control.

### Bacterial OTU richness

Amendments of GAS residue and lime both significantly affected soil bacterial OTU richness (F_11,24_ = 30.336, *P* < 0.001, Figure S2). Specifically, the low level (0.5–2.5 g kg^−1^) amendment of GAS residues gradually elevated bacterial Sobs index, while the high level (25–100 g kg^−1^) amendment of GAS residue and the amendment of lime significantly decreased bacterial Sobs index compared with that of CK. The highest bacterial OTU richness (1908) was at GAS residue amendment at the 2.5 g kg^−1^ content, while the lowest one (1094) was found in lime amendment at content of 50 g kg^−1^. Table S2 reveals the regression analysis results of environmental variables and bacterial OTU richness, and the R and R square of soil TOC and soil nutrients (i.e., NO_3_-N, NH_4_-N, TN) were larger than that of pH. For example, the R and R square of TOC, NO_3_-N, NH_4_-N and TN were 8–30% and 8–69% higher than that of pH.

### Bacterial community structure

**Figure 2:**
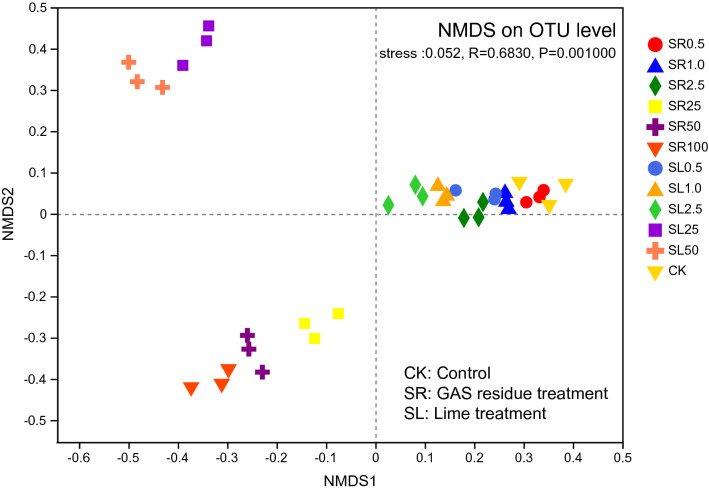
Non-metric multidimensional scaling (NMDS) of soil bacterial communities based on 16S rRNA sequence within plots when GAS residues and lime amended. The numbers 0.5, 1.0, 2.5, 25, 50 and 100 represent the amendment levels (g kg^−1^, n = 3).

The bacterial community structure of the soil samples shifted significantly as a result of the GAS residue and lime amendments, and was clearly distinguishable (Fig. 2, ANOSIM, R = 0.683, *P* = 0.001) among the different amendment treatments. This pattern was confirmed by hierarchical clustering based on OTU level (Figure S3). Specifically, the analysis identified three groups of bacterial community structures (i.e., in the soils amended with high content of lime, soils amended with high content of GAS residue, and soils amended with low content of either lime or GAS residue). Amendment of GAS residue and lime did induce changes in bacterial community structure. The most abundant bacterial phyla detected in soil samples were Firmicutes (8–35%), Proteobacteria (20–22%), Chloroflexi (12–22%), Actinobacteria (8–20%), Cyanobacteria (0.3–16%), Gemmatimonadetes (4–13%), Acidobacteria (1–10%) and Bacterioidetes (1–6%) (Figure S4). More specifically, with the increase in GAS residue amendment from CK to 100 g kg^−1^, the relative abundance of Firmicutes significantly increased from about 8% to 35% (F_6,14_ = 59.84, *P* < 0.001, Figure S4), while that of Chloroflexi significantly declined from about 22% to 12% (F_6,14_ = 36.51, *P* < 0.001, Figure S4). The relative abundance of Gemmatimonadetes and Bacterioidetes significantly raised by 3–6 folds with the amendment of GAS residue from CK to 100 g kg^−1^.

### Soil properties and bacterial community structure

**Figure 3:**
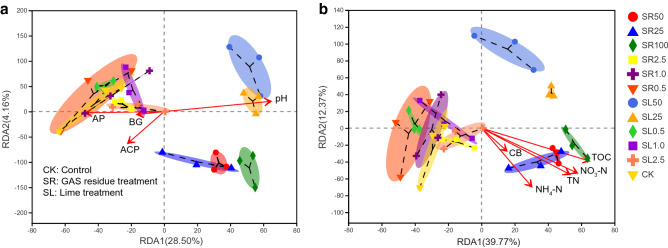
Redundancy analysis (RDA) of bacterial species and environmental factors. *TOC* total organic carbon, *TN* total nitrogen, *NH*_4_*-N* ammoniacal nitrogen, *NO*_3_-*N* nitrate nitrogen, *AP* available phosphorus. *BG* β-1,4-glucosidase, *ACP* acid phosphatase, *NAG* β-1,4-*N*-acetylglucosaminidase, *CB* β-d-cellobiosidase (n = 3).

Based on the results of co-linearity check, environmental variables were classified into two groups, and results of RDA analyses of these two groups to bacterial community structure were shown in Fig. 3. Using RDA analysis (Fig. 3a,b), it was shown that RDA 1 and RDA 2 in Fig. 3a (the environmental variables are pH, ACP, AP and BG ) explained 28.50% and 4.16% of the variations of bacterial community structure, respectively, while that in Fig. 3b (the environmental variables are TOC, TN, NO_3_-N, NH_4_-N and CB) explained 39.77% and 12.37% of the variations of bacterial community structure, respectively. It was observed that CK, low levels amendment of GAS residues and lime clustered, while high levels amendment of GAS residues and lime clustered. As Fig. 3a shows, pH exhibited significant and positive correlations (*R*^2^ = 0.5722, *P* = 0.001) with bacterial community at high levels of lime amendment. Whereas soil nutrients such as TOC, TN, NO_3_-N and NH_4_-N exhibited significant and positive correlations (TOC, *R*^2^ = 0.7120, *P* = 0.001; NO_3_-N, *R*^2^ = 0.6908, *P* = 0.001; TN, *R*^2^ = 0.6881, *P* = 0.001; and NH_4_-N, *R*^2^ = 0.5958, *P* = 0.001) with bacterial community at high levels of GAS residues amendment (Fig. 3 and Table 1).

**Table 1:** The explanatory weight ratio of each dimension.

Index	RDA1	RDA2	R^2^	*p* values
TOC	0.9214	− 0.3885	0.7120	**0.001**
NO_3_-N	0.8699	− 0.4931	0.6908	**0.001**
TN	0.8397	− 0.5431	0.6881	**0.001**
NAG	0.7345	− 0.6786	0.2889	**0.007**
AP	− 0.7133	− 0.7008	0.2797	**0.005**
CB	0.6993	− 0.7148	0.1055	0.162
pH	0.6741	0.7387	0.5722	**0.001**
NH_4_-N	0.6187	− 0.7856	0.5958	**0.001**
BG	− 0.5433	− 0.8395	0.0352	0.545
ACP	0.0254	− 0.9997	0.2740	**0.008**

Bold *p* values represent the significant environmental variables tested by RDA method.

*TOC* total organic carbon, *TN* total nitrogen, *NH*_*4*_*-N* ammoniacal nitrogen, *NO*_*3*_*-N* nitrate nitrogen, *AP* available phosphorus, *BG* β-1,4-glucosidase, *ACP* acid phosphatase, *NAG* β-1,4-*N*-acetylglucosaminidase, *CB* β-d-cellobiosidase.

### Extracellular enzyme activities and bacterial community structure

**Figure 4:**
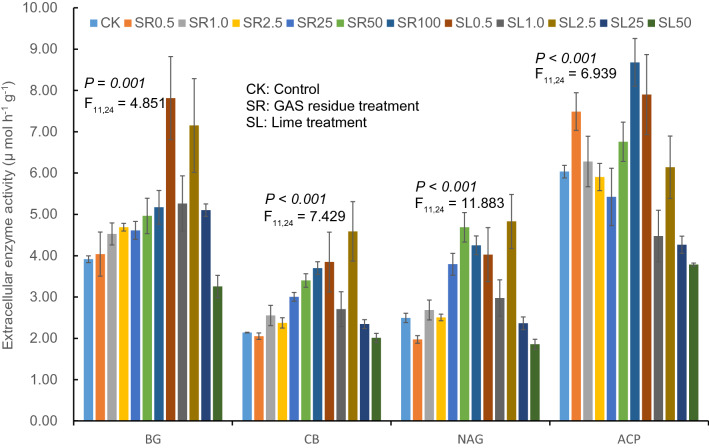
ANOVA results of soil extracellular enzyme activities after incubation. *BG* β-1,4-glucosidase, *ACP* acid phosphatase, *NAG* β-1,4-*N*-acetylglucosaminidase, *CB* β-d-cellobiosidase (n = 3). The numbers 0.5, 1.0, 2.5, 25, 50 and 100 represent the amendment levels (g kg^−1^).

Four enzymes related to C, N and P cycling in soil were determined to reveal the status of soil nutrients and C cycling after the amendment of either GAS residue or lime. ANOVA results suggest that the activities of BG, CB and NAG increased significantly with the increase of GAS residue amendment, while decreased with the elevation of lime amendment (all *P* ≤ 0.001, Fig. 4). Specifically, the activities of BG, CB and NAG increased gradually with the amendment treatment changed and the levels elevated from CK to about SR50–SR100, while these activities declined generally with the treatment changed from SL0.5 to SL50. The highest values of BG, CB and NAG were found in SL0.5 (7.81 m mol h^−1^ g^−1^), SL2.5 (4.59 m mol h^−1^ g^−1^) and SL2.5 (4.83 m mol h^−1^ g^−1^), respectively. However, we observed an “M” shape change of the activity of ACP, specifically the activity of ACP increased from CK to SR0.5, decreased from SR1.0 to SR25, then increased from SR50 to SR100, and then decreased from SL0.5 to SL50. The highest and lowest values of ACP were found in SR100 (8.68 m mol h^−1^ g^−1^) and SL50 (3.79 m mol h^−1^ g^−1^).

## Discussion

### Amendment effect on soil properties

The GAS residue amendment increased soil pH. This may have happened due to the CaCO_3_ in the GAS shell paired with the decomposition of the proteins (from the snail flesh) into amino acids and glucose by soil microbes^47,48^. Soil nitrogen, TN and NO_3_-N content exhibited the highest content when 25 g kg^−1^ GAS residue was added. This was found to be the threshold value prior to which proteins in the GAS residue decomposed or dispersed quickly, but after which anaerobic soils limited both soil microbe activity and the breakdown of proteins into small molecular and inorganic matter, like NH_4_-N, NH_3_ and NO_2_^47^.

### Amendment effect on bacterial OTU richness

Previous studies have proposed that soil pH impacts soil bacterial community structure and diversity^21,49–51^. Fierer and Jackson^26^ found significant correlations between the diversity and richness of soil bacterial communities and soil pH, even across different types of ecosystems, with the highest bacterial diversity being found in neutral soils rather than in acidic soils^26^. Our results are consistent with previous observations^52^, in which bacterial diversity and richness were relatively low in alkaline soils with biochar amendment. We found that soil bacterial OTU richness increased with the addition of either 2.5 g kg^−1^ GAS residue or lime amendments. This is likely because the acidic soil was neutralized to a neutral or slightly alkaline soil. Bacterial OTU richness then declined when more GAS residue or lime were added (≥ 25 g kg^−1^), which may have been due to the effects of different factors such as pH, elevated TOC and nutrient contents. In the SL treatment, a high soil pH likely restricted the growth and proliferation of soil bacteria. This is consistent with the results reported by Xiong et al.^53^, in which soil pH was negatively correlated with soil bacterial OTU richness in alkaline lake sediments across the Tibetan Plateau. Nevertheless, high amounts of soil nutrients in the GAS residue treatment, such as NO_3_-N and NH_4_-N, likely contributed to the decline of bacterial OTU richness. These results differed from the previous work in which the addition of N had no significant effect on bacterial OTU richness, while elevated P did increase bacterial OTU richness marginally^54^.

Regression analysis results revealed that soil TOC and soil nutrients (i.e., NO_3_-N, NH_4_-N and TN) exhibited stronger negative relationships with bacterial OTU richness than that of soil pH (Table S2). Our results contrast with other studies that show positive relationships between soil nutrients and bacterial diversity and richness^54^. This discrepancy may be caused by differences in soil and nutrient type, as well as in the amount of nutrient amendment^26,55,56^. For example, Zhang et al.^57^ reported that addition of NPK nutrients (consisted of urea, KH_2_PO_4_, and K_2_SO_4_) has no significant effects on bacterial diversity or richness in alkaline soil, while the addition of NPK would significantly lower bacterial OTU richness in acidic and near-neutral soils.

### Amendment effect on bacterial community structure

Gemmatimonadetes and Bacterioidetes increased in relative abundance in GAS residue treatments, but not in lime treatments, possibly because Gemmatimonadetes and Bacterioidetes have both been shown to play a role in the soil carbon cycling^58,59^. Amendment of GAS residue (especially high levels, i.e., 50–100 g kg^−1^) increased soil TOC, which likely contributed to the increases in relative abundance of Gemmatimonadetes and Bacterioidetes. Our results contrast with previous studies that found that high soil pH (with biochar amendment) resulted in a decrease in Firmicutes abundance^60^. The present results may reflect the integrated effects of elevated soil pH, C and N (particularly N), because Firmicutes is involved in N cycling^61^. Here, we also noticed that the relative abundance of the bacterial phyla related to N cycling such as Nitrospirae and Actinobacteria significantly and negatively correlated with the elevation of nutrients induced by the amendment of GAS residues (Figure S5), and these results are in line with the finding that N addition decreased both soil microbial diversity and the relative abundance of Actinobacteria and Nitrospirae^62^.

Besides, the significant changes observed in the soil bacterial communities in GAS residue treatments can also be explained by copiotroph and oligotroph mechanisms that are driven by changes in soil nutrients. Copiotrophs and oligotrophs possess an antipodal nutritional requirement as well as totally different utilization mechanisms of C pools. For instance, copiotrophs preferentially consume labile soil organic C pools, while oligotrophs are likely to live in conditions of low nutrient availability because they have higher substrate affinities^63^. In our study, the GAS residue amendment significantly increased the content of soil nutrients (i.e., TN, NO_3_-N,TP and NH_4_-N) and TOC (by as high as 132–912%), and these results may have induced copiotrophic soil conditions that may have benefited copiotrophs and impaired oligotrophs. Our results were similar to previous studies in which bacteria belonging to the Acidobacteria phylum were most abundant in oligotrophic soils, and their relative abundances declined in soils amended with high concentrations of organic C, N and P^54,63^.

### Relationships between soil properties and bacterial community structure

Gemmatimonadetes, Tenericutes, Chlorobi, Firmicutes, Bacteroidetes and Deinococcus-Thermus are all involved in C and N cycling^61^, and the GAS residue amendment induced high levels of C and N, especially at high concentrations (Fig. 1). The negative effects of the GAS residue treatment on Antinobacteria, Cyanobacteria, Nitrospirae, Acidobacteria, Planctomycetes and Verrucomicrobia may reflect sensitivities of some of these groups to higher soil pH. Previous studies have found that some of these groups decrease in abundance after nutrient fertilizer application. For instance, Nemergut et al.^58^ reported that the relative abundance of Verrucomicrobia declined in urea fertilized soil. Jones et al.^64^ reported that although the abundance of Acidobacteria was highly variable among different soil types, strong negative correlations were found with soil pH. Baker et al.^65^ and Xu et al.^66^ reported that Nitrospirae was active in nitrogen cycling, specifically nitrite oxidation, and thus the GAS residue treatment could increase labile nitrogen availability in the soil, which possibly restricted growth of Nitrospirae. Although Prasanna^67^ reported that Cyanobacteria prefer neutral to slightly alkaline pH for optimum growth, we found that the relative abundance of Cyanobacteria decreased in the GAS residue treatment, where an alkaline pH was observed. These results may be caused by the restrictions of elevated soil nutrient levels.

Our results are in line with previous research, in which the estimated soil carbon availability, instead of soil pH, was the main factor strongly correlating with the abundance of Bacteroidetes, Betaproteobacteria, and Acidobacteria^63^. In addition, soil nutrient (N and P) amendments were confirmed to significantly affect soil bacterial community structure^54^. Nevertheless, our results are not consistent with other studies which suggest that pH is a strong predictor of bacterial community structure and diversity^21,49,68^. For example, recently published results suggest that, in GAS residue-treated soil, the soil NH_4_-N content explains more than 48% of the variation in soil microbial properties, while soil pH only explains 8.3% of that variation^19^. In this study, the regression analysis results suggest that the soil TOC and soil nutrients (i.e., NO_3_-N, NH_4_-N and TN) contents exhibited stronger relationship with the change in soil bacterial community structure than did the soil pH in GAS residue amended soil (Table S2).

### Extracellular enzyme activities and bacterial community structure

The amendment treatment induced significantly variations in soil extracellular enzyme activities associated with C, N and P cycling, and the strong correlation between bacterial community structure and enzyme activities may be attributed to changes in liable substrates. Waldrop et al.^69^ reported that bacterial community structure was correlated with BG and ACP activities, and concluded that enzyme activity may reflect the variations between microbial carbon processing and community structure. As Figure S6a shows, significant and negative correlations were observed between bacterial phyla, such as Actinobacteria, Acidobacteriae and Nitrospirae, and enzyme activities, such as CB, NAG and BG. Previous studies have suggested that N addition resulted in significant reductions in soil microbial activity^23^, diversity^24^ and community structure composition^25^ because of increases in C sequestration and/or decreases in soil respiration rates^23^. However, in our study, the enzyme activities significantly increased after the addition of GAS residue, which contains abundant N in the form of proteins and amino acids^18,70^. It is possible that the elevated organic C and N induced by GAS residue treatments promoted hydrolyzation, but suppressed the mineralization of C and N^71,72^. These results are consistent with the results reported by Carreiro et al.^73^, who found that microbes responded to N (in form of NO_3_-N) by increasing cellulase activity. The “M” shape changes of ACP observed here could be attributed to the variations of soil pH and N induced by the amendment of GAS residues and lime. It was observed that the amendment of GAS residues significantly elevated soil N (including TN, NH_4_-N and NO_3_-N) content and pH, while significantly decreased AP content especially at high amendment levels. The input of N would increase the activity of ACP, while the elevation of soil pH would substantially decrease the activity of ACP^74,75^. These results probably indicate that there is a threshold of soil pH, which determines the activity of ACP influenced by addition of N and elevation of soil pH.

### Limitations

Despite the effects of amending GAS residue to acidic and infertile soil on soil properties and bacterial OTU richness as well as bacterial community structure observed in this study, there remain some limitations. For instance, to quantify the usage of GAS residue, the GAS were collected, washed, killed, dried and ground into powder, this process is not cost-effective and is energy-consuming, however, these step-by-step treatments we explored can provide an approach for the possibility and feasibility of amending GAS residue increasing soil fertility and health. Farmers intending to make full use of GAS residue, typically drain the paddy fields affected, and then plough with a rotary tiller to crush and mix the GAS residue into the soil directly. In this way, GAS residues in the paddy fields can be easily used in situ with a low cost and less time input. Our study demonstrates how GAS residue can influence soil health, an important knowledge gap if this agricultural strategy is increasingly adopted.

## Conclusion

Our study suggests that compared to the amendment of lime, the amendment of GAS residue significantly increased soil nutrients (i.e*.,* NO_3_-N and TN) and TOC content, and the amendment of GAS residue could significantly replace soil bacterial community structure and richness. The GAS residue amendment likely induced a copiotrophic environment, in which the relative abundance of copiotrophic bacterial communities increased while oligotrophic bacterial communities declined, and soil exocellular enzyme activities enhanced. Overuse of GAS residue (25–100 g kg^−1^) would induce an anaerobic condition and reduce bacterial OTU richness. In GAS residue amended soil, soil nutrients and TOC rather than pH might be the main factors that are responsible for the changes of bacterial OTU richness and bacterial community structure.

## Supplementary information

Supplementary information.

## Data Availability

The datasets generated during the current study are available from the corresponding author on reasonable request.
