# Supplementary material for: Regulating soil bacterial diversity, community structure and enzyme activity using residues from golden apple snails
Source: Sci Rep. 2020 Oct 1;10:16302. doi: 10.1038/s41598-020-73184-z (PMC7530706; doi:10.1038/s41598-020-73184-z)
Supplement: Supplementary file 1 — Supplementary information. [file 41598_2020_73184_MOESM1_ESM.docx]

**SUPPLEMENTARY MATERIALS**

**SUBJECT AREAS:** Ecology, Environmental Sciences

Correspondence and requests for materials should be addressed to Jiaen Zhang ([jeanzh@scau.edu.cn](mailto:jeanzh@scau.edu.cn) )

**Regulating soil bacterial diversity, community structure and enzyme activity using residues from golden apple snails**

Jiaxin Wang^‡1^, Xuening Lu^‡1^, Jiaen Zhang^1, 2, 3, 4*^, Guangchang Wei^1^ and Yue Xiong^1^

*^1^Department of Ecology, College of Natural Resources and Environment, South China Agricultural University, Wushan Road, Tianhe District, Guangzhou 510642, People’s Republic of China*

*^2^Guangdong Provincial Key Laboratory of Eco-Circular Agriculture, Guangzhou, 510642, People’s Republic of China*

*^3^Guangdong Engineering Research Center for Modern Eco-agriculture and Circular Agriculture, Guangzhou 510642, People’s Republic of China*

*^4^Guangdong Laboratory for Lingnan Modern Agriculture, Guangzhou, 510642, People’s Republic of China*

ORCIDs: Jiaxin Wang: <https://orcid.org/0000-0003-4808-5085> , Jiaen Zhang: <https://orcid.org/0000-0002-2896-7630>

‡ These authors contributed equally to this work.

* Corresponding author: Prof. Jiaen Zhang

College of Natural Resources and Environment, South China Agricultural University

483 Wushan Road, Tianhe District

Guangzhou 510642 People’s Republic of China

Table S1. Total organic carbon (TOC) and soil nutrients including nitrate nitrogen (NO_3_-N), total nitrogen (TN), ammonium nitrogen (NH_4_-N), total phosphorus (TP) and available phosphorus (AP) at different addition levels of the snail residue (SR) and lime (SL) treatments.

|  | **Treatment*** | **TOC (g kg^-1^)** | **NO_3_-N (mg kg^-1^)** | **NH_4_-N (mg kg^-1^)** | **NH_4_-N/NO_3_-N** | **TN (g kg^-1^)** | **TP (g kg^-1^)** | **AP (mg kg^-1^)** |
| --- | --- | --- | --- | --- | --- | --- | --- | --- |
| Background | CK | 16.38±0.56A | 4.20±0.26A | 2.21±0.09A | 0.52±0.01A | 2.20±0.07A | 0.38±0.01A | 30±0.05A |
|  | SR0.5 | 16.35±0.43A | 4.21±0.23A | 2.19±0.12A | 0.52±0.01A | 2.20±0.07A | 0.40±0.02A | 29±0.02A |
|  | SR1 | 16.37±0.48A | 4.22±0.36A | 2.18±0.14A | 0.52±0.01A | 2.19±0.06A | 0.36±0.02A | 29±0.01A |
|  | SR2.5 | 16.29±0.32A | 4.19±0.42A | 2.19±0.13A | 0.52±0.02A | 2.21±0.08A | 0.39±0.03A | 29±0.02A |
|  | SR25 | 16.33±0.26A | 4.19±0.41A | 2.19±0.14A | 0.52±0.01A | 2.20±0.08A | 0.39±0.02A | 28±0.01A |
|  | SR50 | 16.33±0.53A | 4.18±0.24A | 2.20±0.07A | 0.53±0.01A | 2.22±0.11A | 0.40±0.01A | 28±0.03A |
|  | SR100 | 16.36±0.41A | 4.20±0.22A | 2.19±0.11A | 0.52±0.01A | 2.20±0.13A | 0.38±0.01A | 29±0.02A |
|  | SL0.5 | 16.55±0.61A | 4.21±0.23A | 2.20±0.12A | 0.52±0.02A | 2.18±0.14A | 0.37±0.02A | 29±0.02A |
|  | SL1 | 16.46±0.36A | 4.21±0.30A | 2.22±0.11A | 0.53±0.02A | 2.21±0.11A | 0.36±0.02A | 30±0.04A |
|  | SL2.5 | 16.32±0.38A | 4.20±0.33A | 2.19±0.09A | 0.52±0.01A | 2.20±0.07A | 0.37±0.02A | 29±0.03A |
| After treatment | CK | 16.04±0.66e | 3.78±0.43de | 1.82±0.08e | 0.49±0.04ab | 2.19±0.16c | 0.40±0.03d | 26±0.01ab |
|  | SR0.5 | 17.56±0.9cde | 5.51±1.38de | 2.15±0.15cd | 0.45±0.13bc | 2.44±0.16bcd | 0.43±0.04bcd | 28±0.00ab |
|  | SR1 | 17.57±0.62cde | 8.18±0.50c | 2.26±0.09bc | 0.28±0.02cd | 2.40±0.10cd | 0.53±0.05cd | 27±0.02ab |
|  | SR2.5 | 18.46±0.12bc | 12.33±6.06bc | 2.07±0.28cde | 0.21±0.01de | 2.32±0.13cd | 0.51±0.00bc | 27±0.02ab |
|  | SR25 | 26.41±4.43ab | 38.29±0.94a | 2.97±0.06ab | 0.08±0.00f | 5.08±0.42a | 0.55±0.07bcd | 31±0.06ab |
|  | SR50 | 27.96±0.31a | 30.04±1.03ab | 3.40±0.25a | 0.11±0.01ef | 4.87±0.17a | 0.59±0.02bc | 29±0.06ab |
|  | SR100 | 37.57±2.53a | 30.17±1.85ab | 4.86±0.58a | 0.16±0.02ef | 3.84±0.13ab | 0.80±0.07a | 25±0.04b |
|  | SL0.5 | 17.79±0.26cde | 4.40±0.66de | 2.03±0.07cde | 0.49±0.10ab | 2.52±0.07bc | 0.40±0.03d | 29±0.02ab |
|  | SL1 | 16.28±0.78de | 5.33±0.45d | 1.92±0.06de | 0.37±0.04bc | 2.11±0.08d | 0.47±0.08bcd | 31±0.01a |
|  | SL2.5 | 18.21±0.66cd | 2.89±0.52e | 2.11±0.08cd | 0.80±0.19a | 2.27±0.20cd | 0.43±0.03cd | 33±0.03a |

*CK indicates the control; SR indicates the addition of GAS residues; SL indicates the addition of lime; the numbers at the end of SR and SL indicate the addition levels. The results are presented as mean ± SE, the different capital letters in each column represent the significance of background values among different treatments, while the different lowercase letters in each column suggest the significance of treated soils between different treatments according to Duncan's multiple range test.

Table S2. Summary of regression analysis.

| Factor | R | R Square | Adjusted R Square | Std. Error of the Estimate | R Square Change | F Change | df1 | df2 | Sig. F Change |
| --- | --- | --- | --- | --- | --- | --- | --- | --- | --- |
| pH | 0.735^a^ | 0.540 | 0.511 | 259.14272 | 0.540 | 18.762 | 1 | 16 | 0.001 |
| TOC | 0.874^a^ | 0.763 | 0.749 | 185.83393 | 0.763 | 51.597 | 1 | 16 | <0.001 |
| NO_3_-N | 0.764^a^ | 0.583 | 0.557 | 246.55062 | 0.583 | 22.403 | 1 | 16 | <0.001 |
| NH_4_-N | 0.797^a^ | 0.635 | 0.612 | 230.83578 | 0.635 | 27.810 | 1 | 16 | <0.001 |
| TN | 0.804^a^ | 0.646 | 0.624 | 227.23071 | 0.646 | 29.211 | 1 | 16 | <0.001 |
| TOC/TN/NO_3_-N/NH_4_-N | 0.937^a^ | 0.878 | 0.840 | 148.23065 | 0.878 | 23.311 | 4 | 13 | <0.001 |
| pH/TOC/TN/NO_3_-N/NH_4_-N | 0.955^a^ | 0.912 | 0.875 | 130.80120 | 0.912 | 24.889 | 5 | 12 | <0.001 |
| ^a^ Dependent Variable: bacterial OTU richness. | | | | | | | | | |


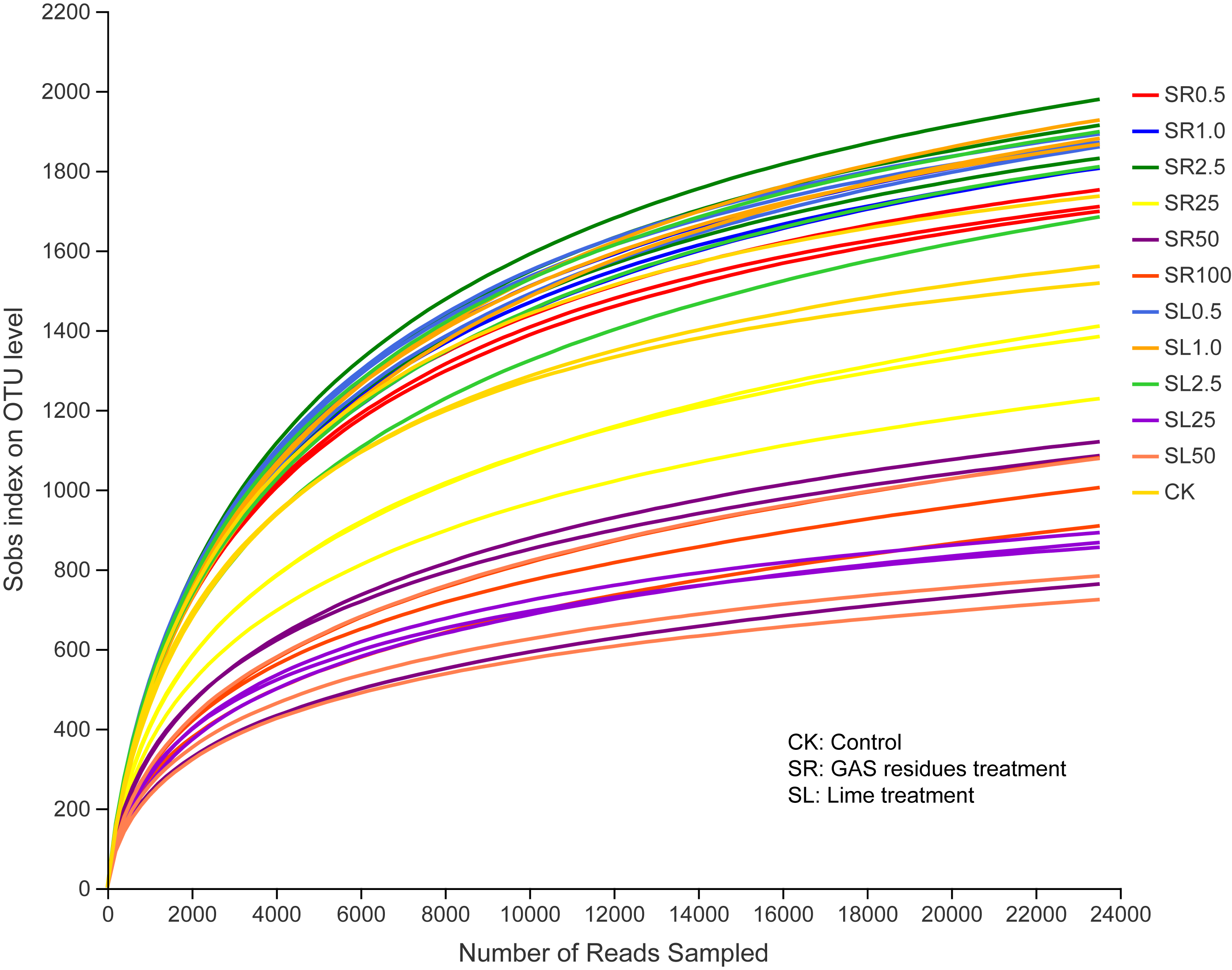
 **Figure S1. Rarefaction curves of bacterial community.** The numbers 0.5, 1.0, 2.5, 25, 50 and 100 represent the amendment levels (g kg^-1^).

**
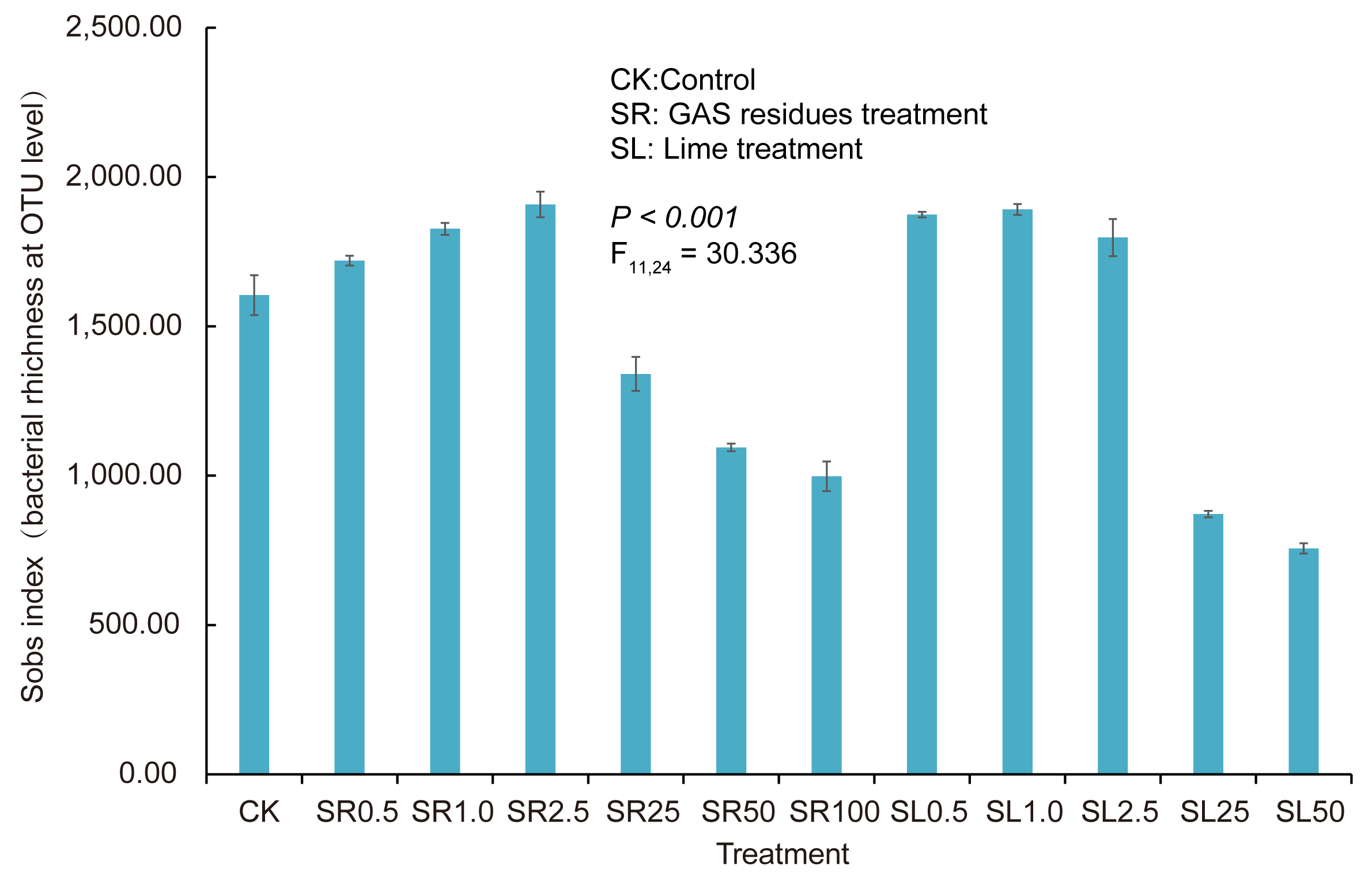
**

**Figure S2.** Variation in the Sobs index of soil bacteria between different amendments. Error bars represent standard errors.


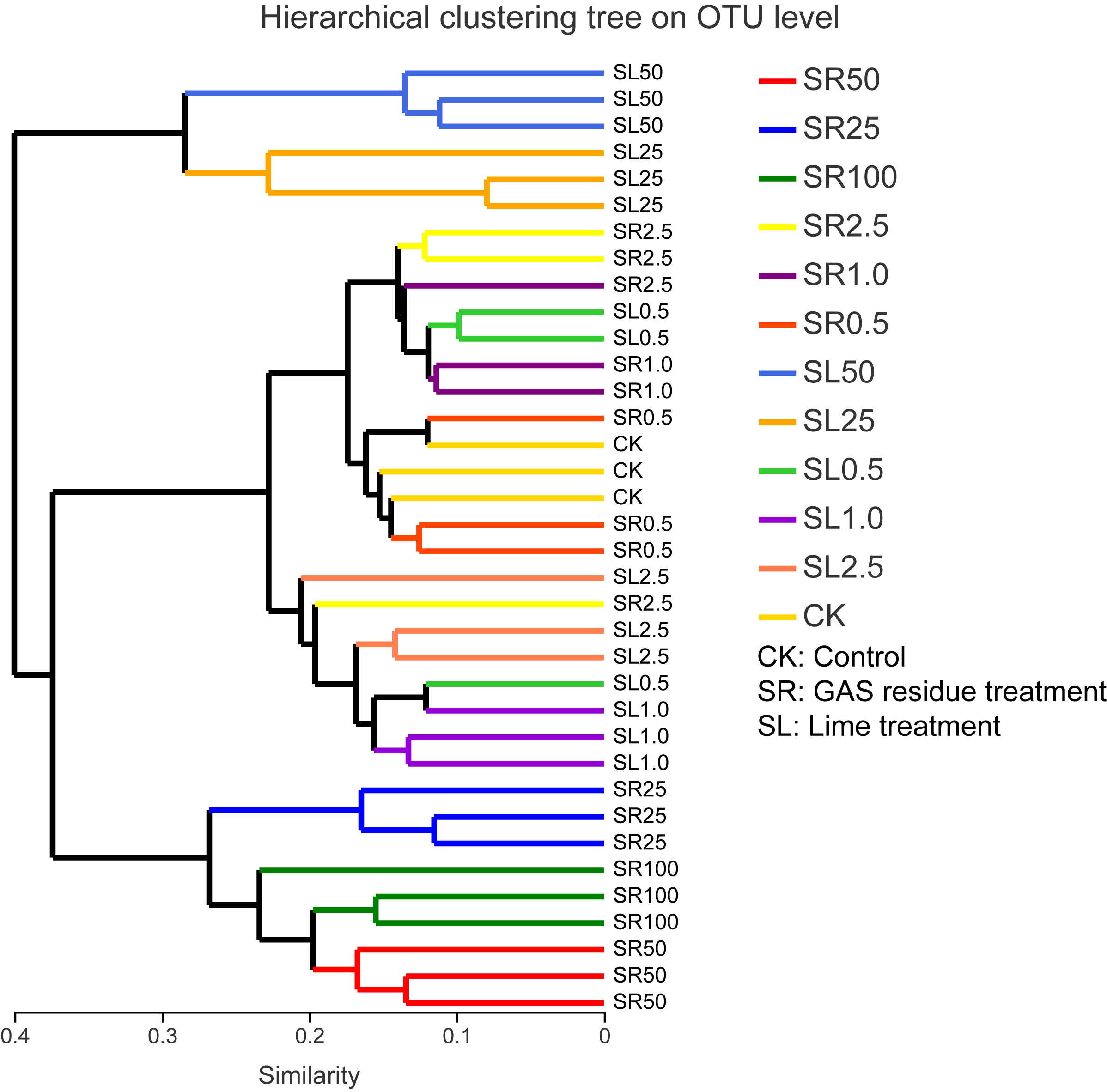
 **Figure S3. Hierarchical clustering of treatments.**

**
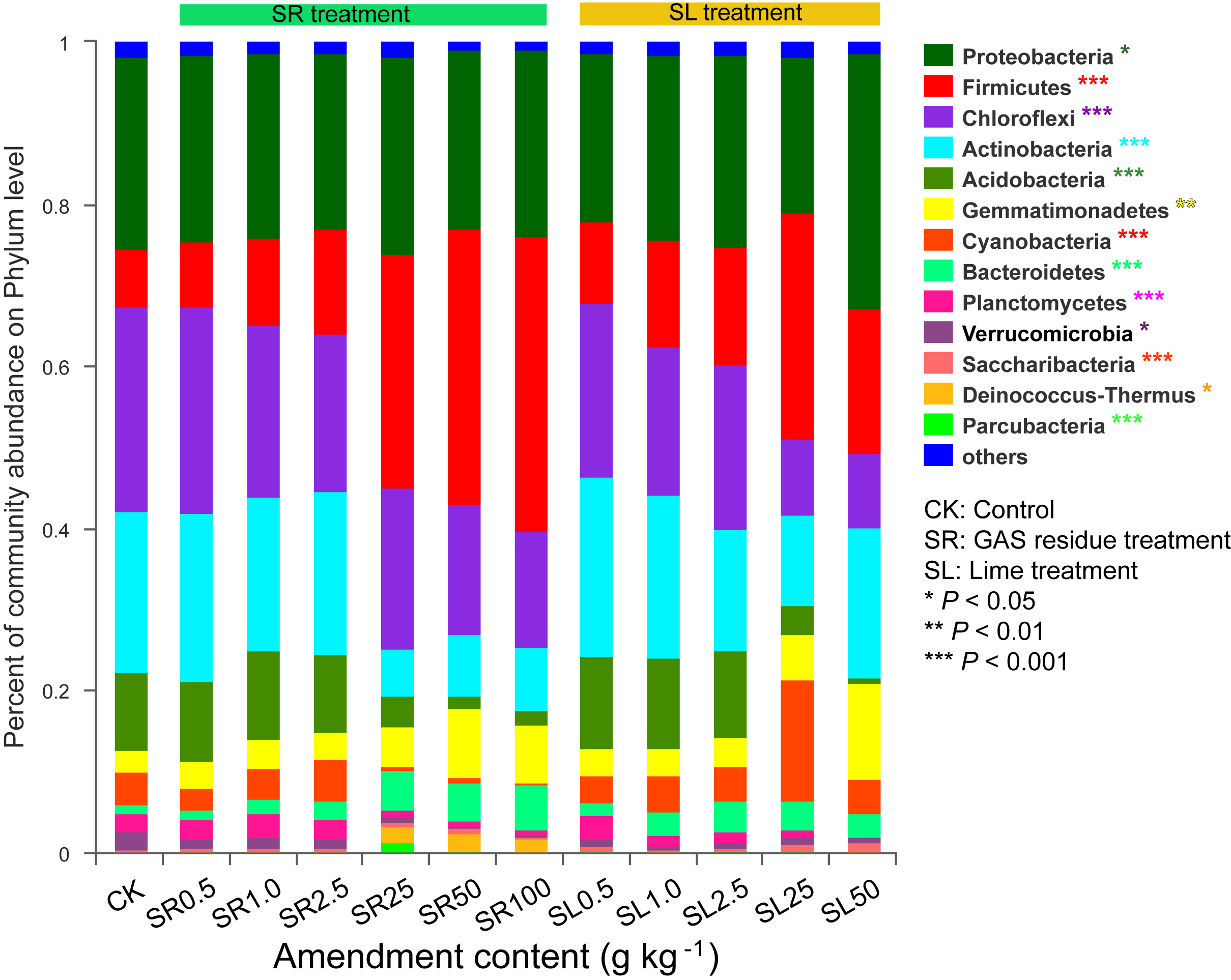
**

**Figure S4. 16S rRNA sequence-based microbial community structure of GAS residue (SR) and lime (SL) amended soils at the bacterial phylum level.** Different colors indicate different phyla as shown in the legend. Asterisks *, ** and *** represent significance at 0.05, 0.01 and 0.001 (n=3).


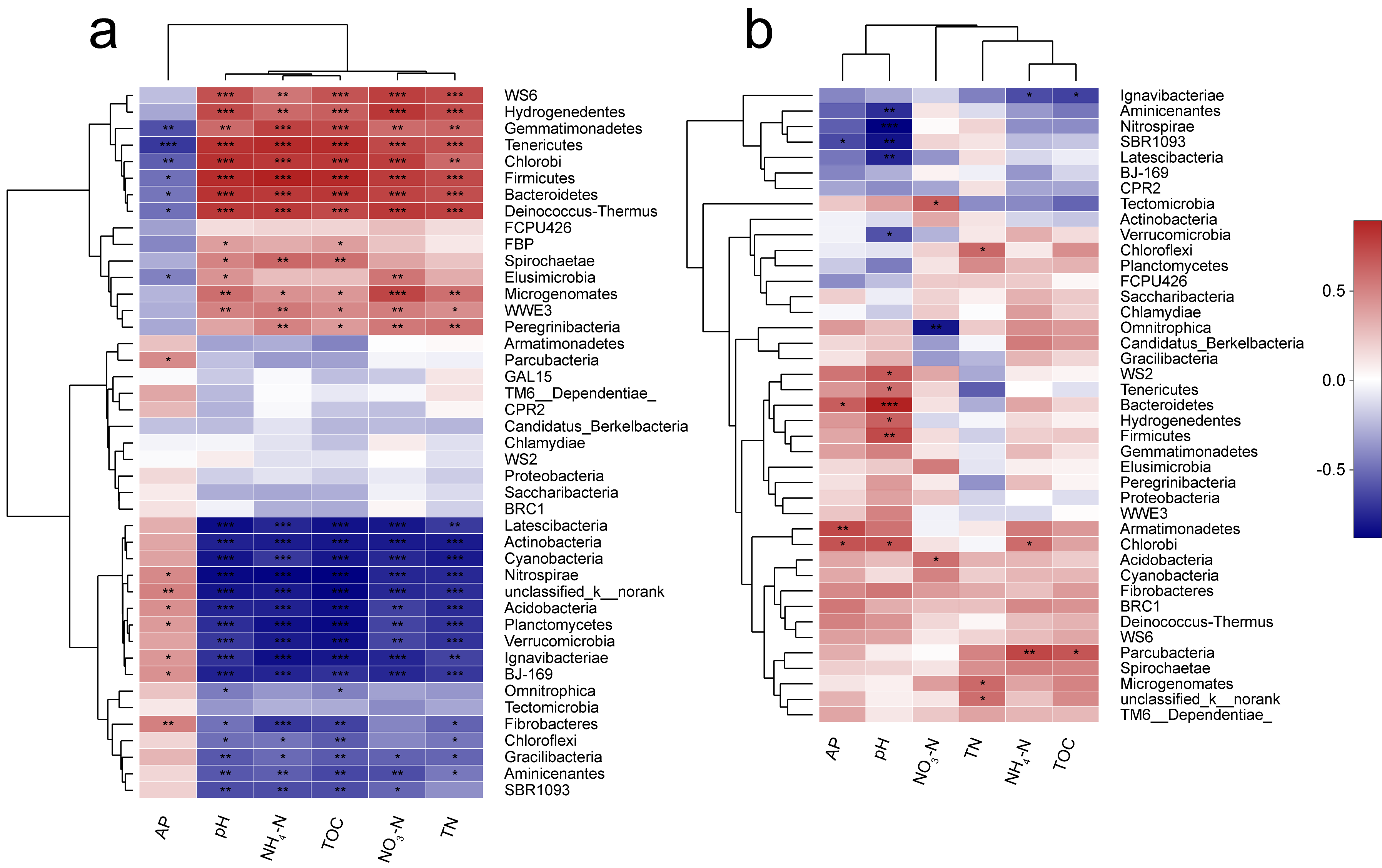


**Figure S5. Spearman’s correlations between soil properties and bacterial community structure at the phylum level after GAS residue (a) and lime (b) amendment.** Blue and red colors represent negative and positive correlations, respectively. TOC represents total organic carbon, TN represents total nitrogen, NH_4_-N represents ammoniacal nitrogen, NO_3_-N represents nitrate nitrogen and AP represents available phosphorus. Asterisks *, **, and *** represent significance at 0.05, 0.01 and 0.001 (n=3).


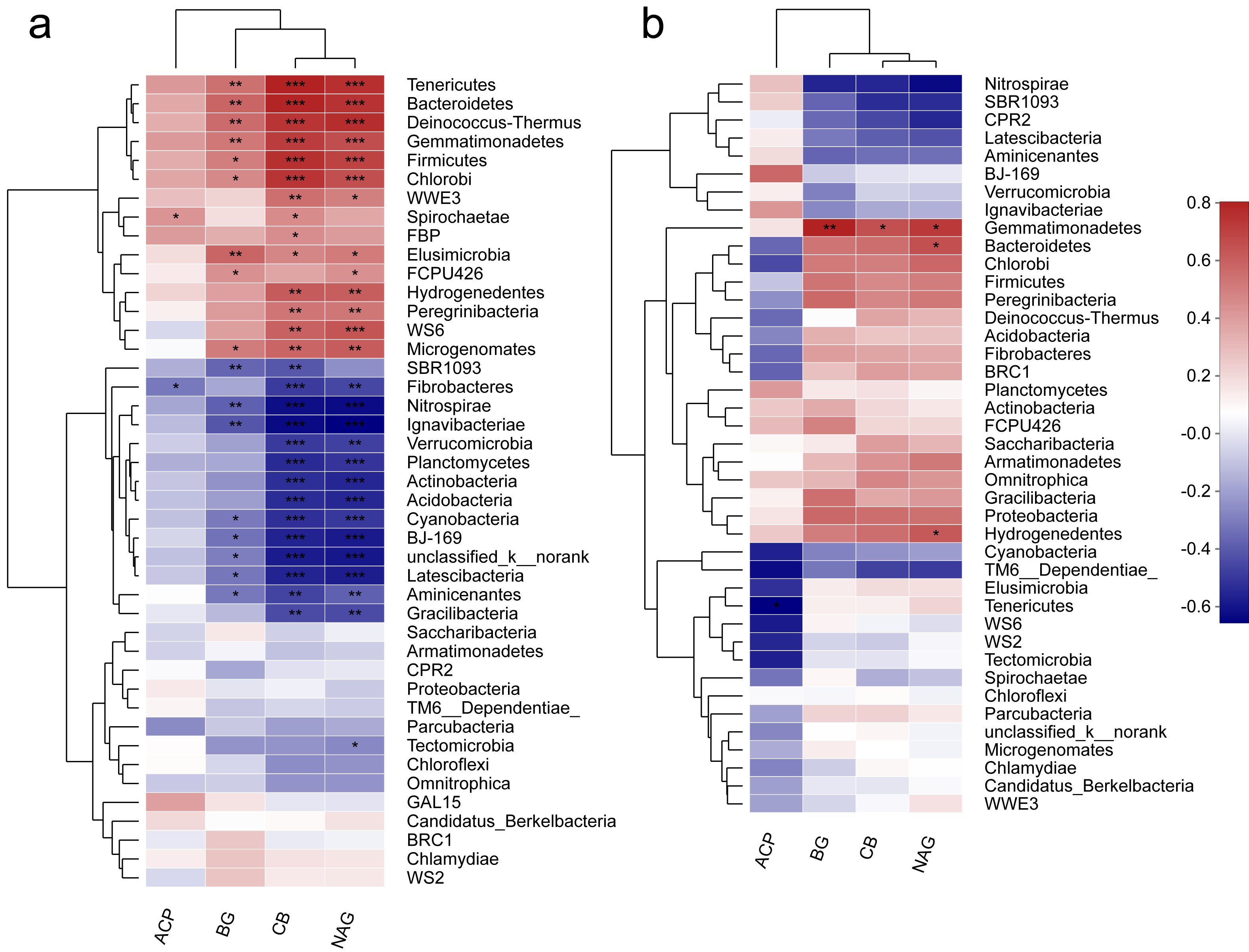


**Figure S6. Spearman’s correlations between soil enzyme activities and bacterial community structure at the phylum level after GAS residue (SR) (a) and lime (SL) (b) amendment.** Blue and red colors represent negative and positive correlations, respectively; β-1,4-glucosidase (BG), acid phosphatase (ACP), β-1,4-*N*-acetylglucosaminidase (NAG) and β-D-cellobiosidase (CB). Asterisks *, ** and *** represent significance at 0.05, 0.01 and 0.001 (n=3).
